# Archives of the Medical Department of the C. S. Army

**Published:** 1890-08

**Authors:** S. H. Stout

**Affiliations:** Ex-Surgeon and Medical Director of Hospitals of the Late Confederate Army and Department of Tennessee; Cisco, Texas


[Sent to Daniel's Texas Medical Journal for publication.]

### THE ARCHIEVES OF THE MEDICAL DEPARTMENT OF THE C. S. ARMY.

---

NEW ORLEANS, LA., July 17th, 1890.

*S. H. Stout, M. D., Cisco, Eastland county, Texas:*

DEAR DOCTOR:—Enclosed please find a copy of my report to his Excellency, General Gordon, commanding United Confederate Veterans. This will explain the nature of the labor in which I am engaged. Can you help me? Do you still retain possession of the hospital reports of the Army of Tennessee? Upon what terms would you place these reports in my hands to aid in my Medical and Surgical History of the C. S. A.?

Will cheerfully bear all express charges. Time is short; I must complete my labors at no distant day.

Hoping you will reply to this at no distant day, I remain, with great respect, truly yours, JOSEPH JONES, M. D.

---

CISCO, TEXAS, July 24th, 1890.

*Prof. Joseph Jones, M. D., New Orleans, Louisiana:.*

DEAR SIR:—Referring to yours of the 17th inst., I have to reply that I positively decline to turn over, on any terms, even temporarily, to you or anyone else, any part of the records in my possession, of the hospital department of the Confederate Army of Tennessee, of which I was Medical Director.

Those records I had the foresight to preserve, with the intention of publishing, after the close of the war, a book or books in the interest of medical science and truthful history.

In the performance of this self-imposed task, I have been engaged for years past, as leisure, amidst the laborious and active practice of my profession, has permitted.

The hospital department, which I directed, was the largest known to have been under the directorship of any one medical officer in either the Confederate or Federal service. At one time its boundaries embraced all the territory occupied by the Confederates between the Savannah and the Mississippi rivers, save only two territorially small directorships in and around Savannah, Georgia, and Mobile, Alabama. The number of medical men, (commissioned surgeons, assistant surgeons, and contract physicians) who served under me, sums up about four hundred. Among these there were very few, if any, who did not, and, if alive, do not now feel proud of the disinterested zeal and efficiency with which they served under my directions. A roster of these, which I have preserved, comprises many names well-known to the medical world.

My high appreciation of the merits of my subordinates in the service as medical men, and of their honorable reputation wherever known, suggested to me the duty of preserving every document which could be used in after times, to illustrate their individual merits and worth, and to preserve the history of their distinguished and unselfishly rendered services.

The records in my possession now, which I have preserved and kept secure, at great cost for transportation and storage, aggregate in weight more than fifteen hundred pounds avoirdupois, and comprise the original morning, weekly and monthly reports of sick and wounded in the hospitals from which I consolidated my reports to the surgeon-general, and to the general commanding the Army of Tennessee; copies of all the general and special orders sent to me from the headquarters of the Army of Tennessee and from the War Department, and my own circulars and reports of medical officers, hospital stewards, detailed men, and matrons; and a vast amount of official and confidential private correspondence, relating to medical service of the department.

In addition to the above, I have documents relating to the construction of hospitals and the transportation of the sick and wounded, which are of great value, and, when published, will prove to be of interest to those engaged in military medical service.

The task of collating and publishing the above records, or such parts of them as shall be adjudged of interest to the cause of medical progress and humanity, and of value to the truthful historian, I voluntarily imposed upon myself, impelled by a sense of duty and considerations above briefly hinted at.

That you or any one else, should expect me to give away or even lend for a time, however short, all or any part of the material on which shall be based whatever publications I may make, touching the hospital department which I directed, in the light of the above statement, you must admit is unreasonable; and it would be unreasonable and unjust, not only to myself but also in a great degree a forfeiture of a trust I hold in behalf of the name and fame of the many faithful and deserving gentlemen who so cheerfully served under my direction, and have, in common with me, an honest pride in the history of the Hospital Department of the Army of Tennessee, which in many of the features of its organization and management, was unique, having no counterpart in respect to those unique features, in either the Confederate or Federal service. Neither yourself nor any other medical officer, who did not serve in the hospital department of the Confederate army and department of Tennessee, can do justice to the distinguished merits and efficient services of those who so cheerfully co-operated with me through my administration as their medical director.

I have endeavored, as briefly as possible, to be explicit, that you and all others may be informed of the grounds on which I base my refusal to grant you or them the use of the valuable records I have so carefully preserved for more than a quarter of a century, for a laudable and unselfish purpose; which purpose would be defeated, were I to part with them before I shall have prepared for the press the naratives and statistics, whose publication I have so long had in view, and hoped to complete at no distant day.

That the information given in this correspondence may possibly reach all whom it may concern, I have taken the liberty of handing the editor of DANIEL'S TEXAS MEDICAL JOURNAL, published in Austin, a copy thereof, with the request that he insert it in his journal, call the attention of the medical and secular press throughout the South to its purport, and ask that it be copied by all newspapers and journals, a considerable moiety of whose patrons may be supposed to be interested in the subject.

Very respectfully

S. H. STOUT, A. M., M. D., LL. D.,

Ex-Surgeon and Medical Director of Hospitals of the Late Confederate Army and Department of Tennessee.

